# Supplementary material for: CO2 Levels Modulate Carbon Utilization, Energy Levels and Inositol Polyphosphate Profile in Chlorella
Source: Plants (Basel). 2022 Dec 27;12(1):129. doi: 10.3390/plants12010129 (PMC9823770; doi:10.3390/plants12010129)
Supplement: Supplementary file 1 [file plants-12-00129-s001.zip › plants-2096370-supplementary.pdf]

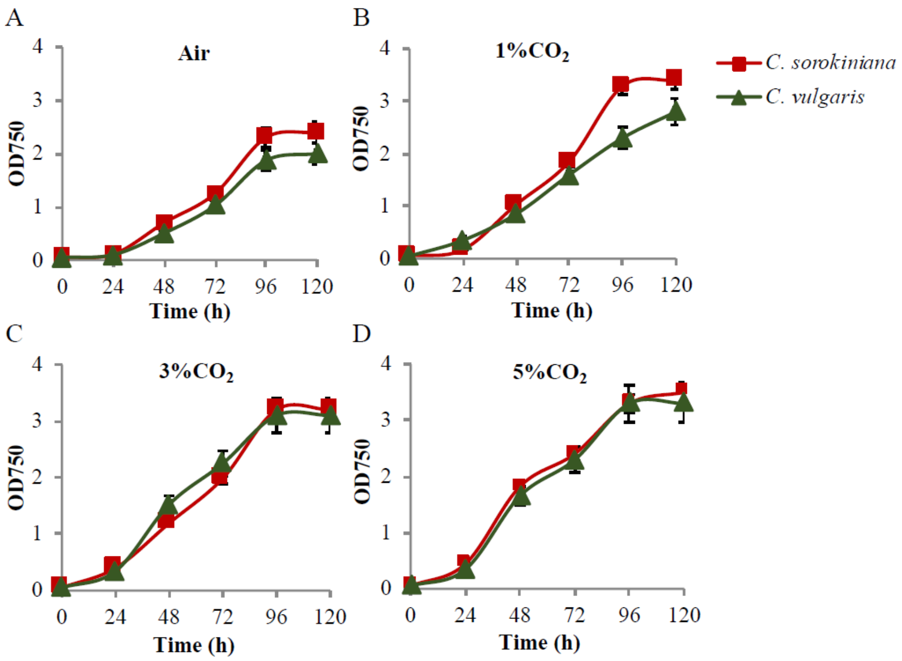

Figure S1. Growth curves from liquid cultures of the indicated strains supplemented with air, 1%, 3% and 5% CO2. Data correspond to the mean± SE of three biological replicates performed in triplicate.

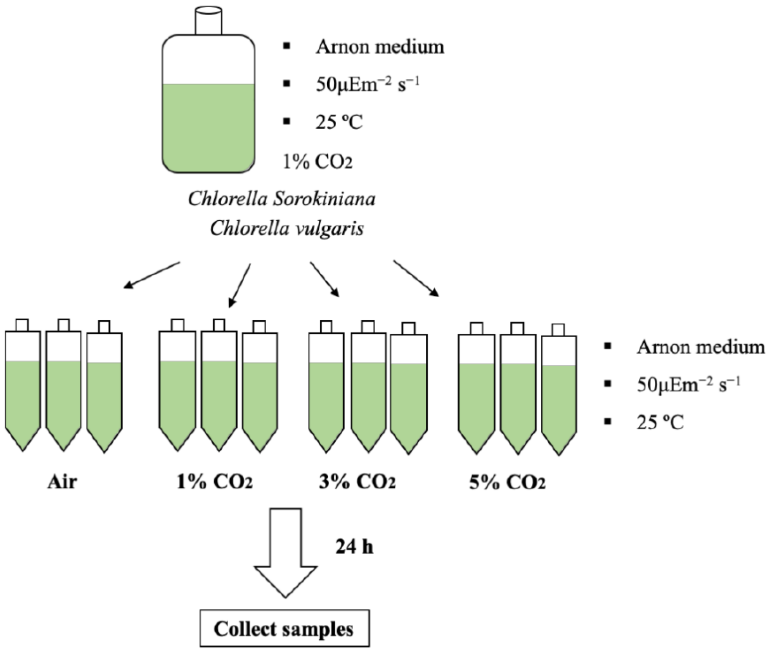

Figure S2. Schematic representation of the experimental data collection.
